# Standard Operating Procedure validity on intramuscular vaccine administration in adults: a methodological study

**DOI:** 10.1590/0034-7167-2022-0692

**Published:** 2023-10-09

**Authors:** Elaine Aparecida da Cunha Lima, Renata Oliveira Caetano, Marisa Dibbern Lopes Correia, Luana Vieira Toledo, Thais Bitencourt Faria, Daniela de Almeida Pereira, Luciene Muniz Braga Daskaleas

**Affiliations:** I Universidade Federal de Viçosa. Viçosa, Minas Gerais, Brazil

**Keywords:** Nursing, Vaccines, Injections, Intramuscular, Validation Study, Process Assessment, Health Care., Enfermería, Vacunas, Inyecciones Intramusculares, Estudio de Validación, Evaluación de Procesos, Atención de Salud., Enfermagem, Vacinas, Injeções Intramusculares, Estudo de Validação, Avaliação de Processos em Cuidados de Saúde.

## Abstract

**Objective:**

to validate a Standard Operating Procedure on the intramuscular vaccine administration technique in adults using high frequency vibration associated with cryotherapy.

**Methods:**

a literature review on intramuscular vaccination practice using a vibration device associated with cryotherapy. Then, a form was created to validate the instrument, detailing the items that were assessed by judges following recommendations in the literature. Judges’ answers were assessed using the Content Validity Index, with items whose index was greater than or equal to 0.80 being validated.

**Results:**

twenty-five nurses participated in validity, identifying judges’ opinion regarding item relevance, clarity and accuracy. Judges validated the instrument, according to the values that remained between 0.88 and 1.0.

**Conclusions:**

the instrument developed and validated is a tool capable of guaranteeing safety and standardizing immunization practice in vaccine rooms.

## RESUMO

**Objetivo:**

validar um Procedimento Operacional Padrão sobre a técnica de administração de vacinas pela via intramuscular em adultos utilizando vibração em alta frequência associada à crioterapia.

**Métodos:**

revisão de literatura sobre a prática de vacinação intramuscular utilizando dispositivo de vibração associado à crioterapia. Em seguida, elaborou-se um formulário para validação do instrumento, com detalhamento dos itens que foram avaliados por juízes seguindo recomendações da literatura. As respostas dos juízes foram avaliadas pelo Índice de Validade de Conteúdo, sendo validados os itens cujo índice foi maior ou igual a 0,80.

**Resultados:**

25 enfermeiros participaram da validação, captando o parecer dos juízes quanto à relevância, clareza e precisão dos itens. Os juízes validaram o instrumento segundo os valores, que permaneceram entre 0,88 e 1,0.

**Conclusões:**

o instrumento desenvolvido e validado é uma ferramenta capaz de garantir a segurança e padronizar a prática de imunização nas salas de vacina.

## RESUMEN

**Objetivo:**

validar un Procedimiento Operativo Estándar sobre la técnica de administración de vacunas intramusculares en adultos mediante vibración de alta frecuencia asociada a crioterapia.

**Métodos:**

revisión de la literatura sobre la práctica de la vacunación intramuscular mediante un dispositivo de vibración asociado a la crioterapia. Luego, se elaboró un formulario para validar el instrumento, detallando los ítems que fueron evaluados por los jueces siguiendo las recomendaciones de la literatura. Las respuestas de los jueces fueron evaluadas mediante el Índice de Validez de Contenido, siendo validados los ítems cuyo índice fuera mayor o igual a 0,80.

**Resultados:**

25 enfermeros participaron de la validación, captando la opinión de los jueces sobre la pertinencia, claridad y precisión de los ítems. Los jueces validaron el instrumento según los valores, que se mantuvieron entre 0,88 y 1,0.

**Conclusiones:**

el instrumento desarrollado y validado es una herramienta capaz de garantizar la seguridad y estandarizar la práctica de inmunización en salas de vacunas.

## INTRODUCTION

Vaccines are among the greatest medical and public health advances^(1)^ and have contributed to vaccine-preventable disease prevention and morbidity and mortality reduction in all age groups in the world^(2)^.

In Brazil, the Brazilian National Immunization Program (PNI - *Programa Nacional de Imunização*), established in 1973, is internationally recognized as one of the most advanced in the world, in addition to being one of the largest public immunization programs. The PNI offers free coverage to all Brazilians and its actions have been expanded over the years, promoting a positive impact on improving the population’s life expectancy^(3)^.

Although vaccines are safe and effective, they can cause acute pain during parenteral administration^(4)^. However, this phenomenon is expected and it is up to the nursing team in the vaccination room to use pain management strategies in their practice^(5)^.

In 2015, the World Health Organization (WHO) took a position on the importance of using viable, accessible, easy-to-perform, lowor no-cost, evidence-based interventions for pain management during vaccination in all countries, regardless of their economic conditions^(6)^.

Recent studies have shown using a high-frequency vibration device associated with cryotherapy to reduce pain during painful procedures^(7-8)^. This technique showed effectiveness mainly with the pediatric population^(9-10)^, proving to be a viable and accessible non-pharmacological intervention^(11)^, but there are few studies to assess the effect in the adult population.

Scientific evidence produces knowledge that underlies nursing actions and practices aimed at caring for individuals’ health. Thus, nursing activities can be linked to technological resources that provide guidance to direct workers and contribute to qualified care^(12)^.

Care protocols can be important resources in health services. Its incorporation can provide quality to health professionals’ work by standardizing the practice, favoring improvements in actions, such as immunization. Thus, nursing team actions in the vaccination room can be standardized through safe, up-to-date procedures based on scientific evidence^(12)^.

The Standard Operating Procedure (SOP) is a type of care protocol that systematically describes each step of the procedures that must be performed by the entire team to ensure the expected results. Moreover, using SOP contributes to training professionals in the correct technique handling and application, helping to synthesize information, clarify doubts, promote the translation of knowledge to improve practice and minimize risks in the face of the procedure. A validated SOP has scientific credibility, being considered a quality management tool in health services, especially those aimed at patient safety through adverse event reduction or eradication^(13)^.

Given the above, this study seeks to validate the standardization of actions by the professionals responsible for the vaccine room during the intramuscular vaccine administration technique, proposing the use of a non-pharmacological measure for pain relief in adults to improve patient care.

## OBJECTIVE

To validate a SOP on the technique of administering vaccines by intramuscular route (IM) in adults using high-frequency vibration associated with cryotherapy.

## METHODS

### Ethical aspects

The study was conducted in accordance with national and international ethics guidelines and was approved by the Research Ethics Committee of the *Universidade Federal de Viçosa*, under registration 5.138.564, whose opinion is attached to this submission, respecting the ethical principles of Resolution 466/2012.

Moreover, the Informed Consent Form (ICF) was obtained from all individuals involved in the study online, with a copy of this electronic document being made available to all participants upon acceptance to participate in the research.

### Design, study location and period

This is a methodological study to validate a SOP content, carried out from December 2021 to March 2022, in the vaccination room of a health unit located at a federal university in the countryside of Minas Gerais, Brazil.

The study took place in two stages. In the first, a literature review was carried out in order to select the best evidence on IM vaccination practice using a high-frequency vibration device associated with cryotherapy for constructing the SOP, and then a form was prepared to validity, with details of items for assessment by judges. The judges invited to participate in content validity were selected through Higher Education Institution websites and the Brazilian National Council for Scientific and Technological Development (CNPq - *Conselho Nacional de Desenvolvimento Científico e Tecnológico*) *Plataforma Lattes*.

### Population and sample, inclusion and exclusion criteria

The instrument assessment was carried out by a group of judges, who were contacted via e-mail individually. In the e-mail, there was a formal invitation presenting the study’s objectives, purpose and development. We selected nurses, with least four years of experience in pain, vaccination and/or public health, professional experience (clinical, teaching or research), article published in an indexed journal, participation for at least two years in research groups, a doctoral and a master’s degree, and residence in the area of interest of the study. It is mandatory to have the criteria of “being a nurse” and “minimum experience of four years in pain, vaccination and/or public health” to participate in the study. Experts who did not respond to the invitation to participate in the study were excluded.

### Study protocol

In order to enable SOP content validity, a scaled questionnaire was prepared, divided into two parts via Google Forms. The first contained questions necessary for characterizing judges according to the criteria proposed by Guimarães et al. (2016)^(14)^, which values individuals’ clinical experience according to the phenomenon under study, assigning a score for classification of experts (junior, master and senior) according to a score of five to 20 points.

Then, the form covered the SOP evaluative items regarding item relevance, clarity and accuracy, separately, through a Likert scale with five levels: 1 - totally disagree; 2 - partially disagree; 3 - indifferent; 4 - partially agree; and 5- totally agree^(15)^. At the end of each evaluative item, there was a space for participants to provide suggestions for improving the instrument.

### Analysis of results and statistics

The agreement among judges’ answers was assessed by the Content Validity Index (CVI), which allows assessing each item of the instrument as well as its entirety. The score was calculated by adding the assessments of the items that received a score of 4 or 5 by judges and was divided by the total number of judges who participated in the survey.

Items whose CVI was greater than or equal to 0.80 were considered validated. The judges could suggest grammatical or writing adjustments for the items^(16)^.

## RESULTS

The sample of judges consisted of 25 nurses, all with recent clinical experience of at least four years in the area of pain, vaccination and/or collective health. Most participants (92%) were female, experienced in research (80%), with articles published in nursing reference journals, with participation in research groups in the areas of pain or immunizations/collective health (36%) and with a doctoral degree in nursing in specific areas (24%) (Table 1).

**Table 1:** Characterization of the sample of judge nurses, 2022 (N=25)

Variable	n	%
Sex		
Feminine	23	92
Masculine	2	8
State in which they work		
MG	24	96
RJ	1	4
Graduation years		
5 to 10 years	7	28
10 to 20 years	14	56
20 to 40 years	4	16
Professional experience		
Research experience with published articles	20	80
Participation in research groups in the area	9	36
Doctorate in nursing in the area		24
Master’s degree in nursing in the area	6	24
Nursing residency in the area	3	12

Then, judges were classified into levels according to Guimarães et al. (2016)^(14)^: 84% received a senior classification, having a score greater than 20 points, or knowledge as much as the junior or master, due to years of experience and 16% received a master classification due to a score between six and 20 points. No judge was considered junior.

SOP content analysis was carried out in stages. First, the judges assessed the header of the instrument, which contained detailed information about the procedure, the person in charge, the supervision, the purpose and the materials required to perform it. In the second session, the actions that should be performed before administering the vaccine were assessed, containing 11 items, explaining the steps to be followed in the reception room. In the session related to preparing the vaccine in the application room, 10 items were assessed. All items in the referred sessions received a CVI 1.0 among experts, receiving suggestions only for adapting the wording of items 1.9 and 1.11 (Chart 1).

**Chart 1:** Judges’ assessment of relevance, clarity and accuracy for heading, objective, materials, reception room and application room: preparation of the Standard Operating Procedure, Viçosa, Minas Gerais, Brazil, 2022

Items	Relevance - CVI	Clarity - CVI	Accuracy - CVI
Heading - Procedure: administration of vaccines packaged in a vial with a rubber stopper, intramuscularly, in the deltoid muscle, within the basic health unit, using a portable high-frequency vibration device associated with an ice pack for 30 seconds before and during vaccine administration. Responsible: nursing staff. Supervision: nurses.	1.0	1.0	1.0
Objective - Guide nursing professionals on the technique of administering vaccines intramuscularly in the deltoid region, using a portable high-frequency vibration device associated with an ice pack.	1.0	1.0	1.0
Necessary materials - 01 high frequency vibration external plastic device; 01 frozen gel bag; 03 AAA batteries; 01 stopwatch; 01 pen; 01 “rat-tooth” tweezers or staple extractor; 1.0 ml or 3.0 ml disposable plastic syringes; disposable needles 25x8.0 dec/mm; 25x7.0 dec/mm; 25x6.0 dec/mm; and 20x5.5 dec/mm; cotton; 70% alcohol%; sterile absorbent drape; 01 cutting drill box; 01 stainless steel tray; procedure gloves.	1.0	1.0	1.0
**Before administering the vaccine - reception room**			
1.1 Ensure minimum waiting time for service.	1.0	1.0	1.0
1.2 Assess users’ current state of health.	1.0	1.0	1.0
1.3 Assess users’ vaccination history and ensure the correct vaccine is offered	1.0	1.0	1.0
1.4 Inform about the vaccine administration, the benefits and risks of receiving or not the vaccine.	1.0	1.0	1.0
1.5 Investigate history of severe reaction after receiving vaccine or if they are aware of any allergy to vaccine components.	1.0	1.0	1.0
1.6 Advise on the possible occurrence of post-vaccination local and systemic adverse events such as local pain, redness, swelling, fever, body pain, diarrhea and malaise.	1.0	1.0	1.0
1.7 In cases of local and systemic adverse events after vaccination, advise to : use a cold compress at the vaccine application site to alleviate local pain, redness and swelling; rest at home, in a well-ventilated environment, drink liquids for oral rehydration, and if necessary, use analgesics prescribed by a doctor to alleviate fever, body pain, diarrhea and malaise; seek medical attention if the post-vaccination local and systemic adverse event(s) do not improve or worsen; communicate the symptoms presented to the vaccination service to make the notification.	1.0	1.0	1.0
1.8 Provide an opportunity for users and/or companion/guardian to ask questions and clarify doubts.	1.0	1.0	1.0
1.9 Obtain consent from users or companion/guardian to vaccinate.	0.96	0.96	0.96
1.10 Write down the name of the vaccine in the vaccination booklet, date of administration with day, month and year, batch, validity of the vial, manufacturing laboratory, health unit and leave space for the vaccinator to sign, schedule return of the next dose, if indicated.	1.0	1.0	1.0
1.11 Register the administered dose in the information system in force in the Brazilian National Immunization Program.	1.0	0.96	1.0
**Application room: preparation of vaccine administration**			
2.1 Clean the bench, chairs and stretcher with 70% alcohol.	1.0	1.0	1.0
2.2 Clean the hands with soap and water, according to the institution’s standard operating procedure for hand hygiene.	1.0	1.0	1.0
2.3 Welcome users and close the door to ensure privacy.	1.0	1.0	1.0
2.4 Organize, check the integrity and expiry date of all necessary material.	1.0	1.0	1.0
2.5 Check the notes on the vaccine card, confirming the vaccine to be administered and the data of users who will receive it.	1.0	1.0	1.0
2.6 Place users in a sitting position or in a supine or lateral decubitus position (if necessary).	1.0	1.0	1.0
2.7 Explain the procedure for administering the vaccine and clarify doubts.	1.0	1.0	1.0
2.8 Give users the opportunity to choose the right or left deltoid for administering the vaccine, avoiding places with scars, stains, tattoos and injuries.	1.0	1.0	1.0
2.9 Fully expose the upper limb from the shoulder to the elbow. If necessary, ask to remove the blouse, maintaining privacy.	1.0	1.0	1.0
2.10 Assess skin dirtiness, if necessary, wash the site where the vaccine will be administered with soap and water or use cotton soaked in 70% alcohol for 30 seconds in a single direction. Wait 30 seconds until the alcohol evaporates completely.	1.0	1.0	1.0

In the next session, referring to the technique to be performed during vaccine administration, experts assessed 27 items that contained the steps to be followed for the correct vaccine administration. The agreement was 100% in the assessed criteria, and only the items referring to the Z technique received 0.88 of CVI agreement. For these, experts suggested assessing the use of the referred technique in vaccine application.

Finally, the last session contained 10 items related to post-vaccination actions, which should be carried out by the nursing team at the end of individuals’ immunization. The items referring to the completion of the procedure received a concordance score of 1.0 CVI among experts, with only items 4.9 and 4.10 (0.92 and 0.96 CVI, respectively) receiving brief suggestions for wording adequacy (Chart two).

**Chart 2:** Judges’ assessment regarding relevance, clarity and accuracy for application room: technique and application room: post vaccination of the Standard Operating Procedure, Viçosa, Minas Gerais, Brazil, 2022

Application room: technique	Relevance - CVI	Clarity - CVI	Accuracy - CVI
3.1 Clean hands with 70% alcohol-based antiseptic, when there is no visible dirt, or wash with soap and water, in cases of dirt. If necessary, put on procedure gloves (presence of open lesions on the hands or risk of contact with body fluids), changing and cleaning hands between users.	1.0	1.0	1.0
3.2 Choose syringe size according to the volume of the vaccine to be administered and choose needle length and gauge according to users’ biotype.	1.0	1.0	1.0
3.3 Check vaccine name, liquid integrity and expiration date.	1.0	1.0	1.0
3.4 Show users the vial and read along with them the name of the vaccine to be administered and the expiration date.	1.0	1.0	1.0
3.5 Remove the seal from the vaccine vial with the aid of rat-tooth forceps or the staple extractor and clean the rubber of the vial with dry cotton.	1.0	1.0	1.0
3.6 Attach a 25x7.0 dec/mm, 25x8.0 dec/mm needle to the syringe and introduce it into the vial.	1.0	1.0	1.0
3.7 Homogenize the liquid, rotating the flask in one direction and without producing foam, until a uniform suspension is obtained.	1.0	1.0	1.0
3.8 Place the syringe in a vertical position, at eye level, aspirate the liquid corresponding to the dose to be administered and eliminate the air with the needle still inside the vial, immediately before its application.	1.0	1.0	1.0
3.9 Replace the 25x7.0 dec/mm or 25x8 dec/mm needle with a 25x6.0 dec/mm or 20x5.5 dec/mm needle, according to users’ biotype and keep it protected.	0.96	0.96	0.96
3.10 Pack the syringe in the thermal box.	0.96	0.96	0.96
3.11 Position the upper limb aligned with the body with the shoulder relaxed and the forearm on the abdomen.	1.0	1.0	1.0
3.12 Locate the deltoid muscle by identifying the acromion, mark 3 cm below the acromion and draw an imaginary triangle with the base facing upwards.	1.0	1.0	1.0
3.13 Take the ice pack from the freezer, attach it to the back of the portable high-frequency vibration device and place it in contact with the skin, in the center of the imaginary triangle, where the vaccine will be administered. Fix the vibration device associated with the ice pack with the tourniquet that comes with said device, turn on the vibration button and time 30 seconds. After that time, with the device still on, slide the device about 2 cm above the vaccine administration site.	1.0	1.0	1.0
3.14 Administer the vaccine using the Z technique.	0.96	0.96	0.96
3.15 Introduce the needle into the deltoid muscle in the center of the imaginary triangle, at a right angle (90º) with lateral bevel.	1.0	1.0	1.0
3.16 Do not vacuum.	0.96	0.96	0.96
3.17 Maintain Z technique while administering the vaccine rapidly.	0.88	0.88	0.88
3.18 Withdraw the needle with a quick, firm movement and undo the Z technique.	0.88	0.88	0.88
3.19 Activate the safety system on the needle, if applicable.	1.0	1.0	1.0
3.20 Place dry cotton and maintain light pressure on the vaccine administration site.	1.0	1.0	1.0
3.21 Disconnect and remove the vibration device associated with the ice pack.	1.0	1.0	1.0
3.22 Do not rub the vaccine administration site.	1.0	1.0	1.0
3.23 Observe if there is bleeding at the vaccine administration site, if necessary, maintain compression until the bleeding stops.	1.0	1.0	1.0
3.24 Observe user reactions, be aware of immediate adverse events.	1.0	1.0	1.0
3.25 Dispose of the syringe with the needle in the sharp puncture box.	1.0	1.0	1.0
3.26 Remove the cotton and dispose of it in the infectious waste.	1.0	1.0	1.0
3.27 Apply absorbent covering over the vaccine administration site.	1.0	1.0	1.0
**Application room: post-vaccination**			
4.1 Dispose of the syringe and needle packaging in the common trash.	1.0	1.0	1.0
4.2 Dispose of the bottle in an appropriate place, in accordance with legislation.	1.0	1.0	1.0
4.3 Clean the hands with soap and water, according to the institution’s standard operating procedure for hand hygiene.	1.0	1.0	1.0
4.4 Sign the name in full, legibly, on the vaccination card and return it to users.	1.0	1.0	1.0
4.5 Instruct users to remain seated in the unit’s premises for 15 minutes for observation.	1.0	1.0	1.0
4.6 Clarify doubts.	1.0	1.0	1.0
4.7 Dismiss users.	1.0	1.0	1.0
4.8 Disinfect the portable high-frequency vibration device with 70% alcohol.	1.0	1.0	1.0
4.9 Disinfect the gel bag with 70% alcohol and return it to the freezer.	0.92	0.92	0.92
4.10 Clean the hands with soap and water, according to the institution’s standard operating procedure for hand hygiene.	0.96	0.96	0.96

In general, judges’ CVI level of agreement regarding the instrument’s items remained between 0.88 and 1.0.

## DISCUSSION

Content validity studies aim to improve, refine and improve diagnoses, interventions and results from clinical practice. Thus, it is also used to validate instruments that standardize care, seeking to standardize language, improve care and fill gaps in practice. Thus, the literature has shown that content validity studies are constantly evolving, as is the nursing process^(17)^.

In this study, we chose to validate a SOP on the IM vaccine administration technique using non-pharmacological intervention based on the instrument assessment by a group of judges. Regarding content validity studies, the literature shows strong scientific evidence on the participation of experts in the instrument assessment, always taking into account the experience and qualification of the members of this committee^(16)^. Thus, the criteria proposed by Guimarães et al (2016)^(14)^ were adopted for the inclusion of judges, valuing their professional experience according to the study phenomenon.

Judges’ profile corroborates the socio-demographic aspects analyzed by the study by Machado et al (2016)^(18)^, with most judges residing in the Southeast and mostly female. The age and stage of professional life of the sample of judges reflects the reality of the Brazilian nursing profile, corroborating the findings of research by Machado et al. (2016)^(18)^, in which 28% of the sample is present in the professional training phase (26 to 35 years old) and 72% in the professional maturity phase (36 to 50 years old). Allied to this, judges’ working time and professional experience, for the most part, were between 10 and 20 years in the areas of pain, vaccination and/or collective health, which demonstrates a sample composed of qualified and specialized nurses within the specific areas of interest of this study.

Thus, with their cognitive skills, techniques and practices consolidated in the areas of pain, vaccination and/or public health, the judges received a senior (84%) and master (16%) classification, according to criteria proposed by Guimarães et al (2016)^(14)^. The adopted classification criteria value clinical experience over academic experience, denoting the strength of the opinion of this group of experts. Judge characterization by levels also made it possible to better understand experts’ profile, confirming the professional phase in which they were and highlighting the specialization in the areas of this study.

The content validity procedure made it possible to identify judges’ opinion regarding the SOP assessment items, such as item relevance, clarity and accuracy, separately, which subsidized the reformulation, mainly grammatical, of some items. According to the stipulated CVI of 0.80^(16)^, it was observed that the judges considered the instrument’s items adequate, therefore, the instrument was validated.

Despite the content validity obtained through CVI greater than or equal to 0.80, experts expressed some suggestions for improving the clarity and accuracy of some SOP items.

In order to improve the instrument, items 1.9 and 1.11 had their wording improved to: “Obtain consent from users or companion/guardian to vaccinate” and “Register the administered dose in the information system in force in the Brazilian National Immunization Program”, respectively, aiming at improving item clarity and accuracy.

One of the suggestions for improving item 3.9 was to modify the wording of “Exchange the 25x7.0 dec/mm needle for a 25x6.0 dec/mm or 20x5.5 dec/mm needle and keep it protected” to: “Replace the 25x7.0 dec/mm or 25x8 dec/mm needle with a 25x6.0 dec/mm or 20x5.5 dec/mm needle, according to users’ biotype and keep it protected”, to make it clearer and need the information. Changing the needle before IM injections is a factor that also helps to reduce injection pain^(19)^. Item 3.10, on the other hand, received only one comment from an expert who does not adopt such action, of packaging a syringe in a thermal box, in his daily practice, alleging risks of contamination. In view of this, it is worth noting that, in order to use the vibration equipment, associated with the ice pack, when placed on the patients’ arm, a timer must be set for 30 seconds before applying the immunizer, which justifies, in this case, the need to pack the syringe in a thermal box, aiming at greater security in maintaining the immune system at a cold temperature. Thus, the installation of the thermal box must comply with the institution’s SOP for this purpose. In this thermal box assembly SOP, it should be described to use a material/container in order to store the vials and syringes of vaccines so that they are not in direct contact with artificial ice plates, avoiding their freezing^(20)^. Furthermore, the needle of the syringe containing the vaccine, after being aspirated, will be protected with its plastic cap and packed in the thermal box (inside the container) to carry out the Buzzy installation and timing procedure.

In addition to the above, the device’s manual states that the ice pack (which corresponds to a bee’s wings) must be kept in the freezer and removed only at the time of use. Immediately after removal from the freezer, it must be coupled to the back of the vibration device, fixed on the deltoid, pressing the button to start the vibration, timing 30 seconds, moving the device coupled to the ice pack for about 2 to 3 cm proximal and administer the vaccine. After administration, remove the device, turn it off, uncouple the ice pack, disinfect both with 70% alcohol and return the ice pack to the freezer^(21)^.

Despite items 3.14, 3.16, 3.17 and 3.18, which refer to the step by step of the IM administration Z technique, receiving CVI greater than 0.80, some experts questioned the use of that technique in vaccine administration. The recommendations were: “Assess the use of the Z technique in vaccine administration” in the scientific literature. In view of this, the literature shows, according to a systematic review and meta-analysis performed by Zeyrek et al (2019)^(19)^, that the Z technique is an alternative for reducing pain during IM injections. Additionally, there is a recommendation that there is no pleat before application in deltoid, since, according to the authors, skin and muscle grouping can impair the actual IM insertion of the vaccine, by increasing the subcutaneous tissue, especially in obese people^(22)^. Thus, using the technique in question and the high-frequency vibration device associated with cryotherapy would be a factor both in reducing pain in patients and in the actual vaccine IM introduction, as recommended by the manufacturer.

In addition, final items 4.9 and 4.10, which concern disinfection with 70% alcohol of the ice pack and hand hygiene with soap and water at the end of the procedure, received suggestions for changing item wording. However, after critical analysis of the suggestions, it was decided to maintain the original wording.

Thus, the SOP instrument was restructured and is now suitable for carrying out the IM vaccine administration procedure using vibration equipment associated with cryotherapy. This initiative aims to enable professionals to guide the implementation of a non-pharmacological technique for pain relief in patients, as recommended in the literature^(8,10,23)^. It is highlighted that the use of pain reduction strategies before the application of injectables should be taken by professionals with a focus on the client as a unique being who wants to receive this type of intervention^(8,23)^.

### Study limitations

As a study limitation, the instrument size stands out, a factor that generated low participants’ compliance, which is very extensive and available online, which generated a large number of experts who did not respond to the invitation to participate in the study, being carried out 100 invitations via email and only 28 participation answers. Moreover, most of participating experts lived in southeastern Brazil. Thus, there may be differences in the practices adopted by these judges compared to others from other regions, not reflecting the national territory.

### Contributions to nursing, health or public policy

During the literature review, no studies with methodological rigor were identified on the subject that presented all the steps for administering vaccines by IM route in the deltoid. Thus, this study stands out, as it presents contributions to the field of nursing, presenting a developed and validated instrument. It is a tool capable of guaranteeing safety and standardizing nursing professionals’ work in the vaccination room, enhancing the implementation of immunization practices consistent with the quality of care provided by the nursing team.

## CONCLUSIONS

The study achieved the proposed objective, as it was possible to validate the SOP with experienced judges. The nurses who participated in this research recognized the importance of standardizing IM vaccine application using non-pharmacological measures for pain relief, contributing to improving the instrument.

The instrument was elaborated in the light of literature and the results supported by scientific evidence and judges’ professional experience. It is hoped that this study and the validated instrument will contribute to nurses’ practice in immunization rooms and show the importance of implementing the SOP and standardizing the nursing team actions in the different health care environments.

## Data Availability

*https://doi.org/10.48331/scielodata.OUZVJI*
